# Case Report: Parasomnia Overlap Disorder Induced by Obstructive Sleep Hypopnea Apnea Syndrome: A Case Report and Literature Review

**DOI:** 10.3389/fnins.2020.578171

**Published:** 2020-12-10

**Authors:** Yun Sun, Jie Li, Xinjun Zhang, Qingyan Jiao, Shutong Yang, Lijie Ji

**Affiliations:** ^1^Department of Sleep Medicine, Tianjin Anding Hospital, Tianjin, China; ^2^Institute of Mental Health, Tianjin Anding Hospital, Tianjin, China

**Keywords:** obstructive sleep apnea hypopnea syndrome, dreams, nightmare, parasomnia overlap disorder, REM sleep behavior disorder, sleep terrors

## Abstract

Obstructive sleep apnea hypopnea syndrome (OSAHS) and parasomnia overlap disorder (POD) are types of sleep disorders. When the symptoms of both conditions coexist, the POD symptoms are most likely caused by OSAHS. In these cases, the symptoms of POD will be relieved when OSAHS is effectively treated. We refer to these cases as symptomatic POD (related to OSAHS), which differs in pathophysiology, complications, and treatment from idiopathic POD. It is important to note that the treatment for idiopathic POD may aggravate the symptoms of OSAHS. In this case, we used video polysomnography (v-PSG) on a POD patient with suspected OSAHS to distinguish idiopathic POD from symptomatic POD, to inform the appropriate treatment course. The video results and clinical features lead us to diagnose symptomatic POD, and we treated the patient with auto-set continuous positive airway pressure to address their OSAHS. This course of treatment resolved all POD-related symptoms. Here, we discuss this case and review the relevant literature. This report highlights the importance of the use of v-PSG in the clinical diagnosis, differential diagnosis, and subsequent treatment of POD.

## Introduction

Obstructive sleep apnea hypopnea syndrome (OSAHS) is a seriously underestimated chronic disease. Untreated patients have a significantly increased risk of cardiovascular disease (Salman et al., 2020), metabolic diseases (Jehan et al., 2018), neurocognitive disorders, and traffic accidents and observe a decline of both labor capacity and life quality (Kapur et al., 2017; Xiao, 2019; Gottlieb and Punjabi, 2020). Parasomnias are a category of sleep disorders that involve abnormal movements, behaviors, emotions, perceptions, and dreams that occur while falling asleep, when sleeping, between sleep stages, or during arousal from sleep (Howell, 2012). Obstructive events induce recurrent sleep fragmentation and intermittent desaturations in patients with OSAHS (Mendonca et al., 2019), which may trigger various parasomnias, including nightmares (BaHammam and Almeneessier, 2019).

Parasomnia overlap disorder (POD) occurs when patients demonstrate features of both non-rapid eye movement (NREM) sleep parasomnias (confusional arousals, sleepwalking, sleep terrors, and sleep sex) and rapid eye movement (REM) sleep parasomnias [REM sleep behavior disorder (RBD) and nightmares] (Dumitrascu et al., 2013; Irfan et al., 2017; Xu et al., 2019). REM sleep behavior disorder is a parasomnia characterized by repeated episodes of vivid, disturbing dreams enactment behavior and REM sleep without atonia, detected during polysomnography (PSG) recording and manifested as increased phasic and/or tonic muscle activity on electromyogram channels (Haupt et al., 2013). REM sleep behavior disorder may be idiopathic or symptomatic (secondary), strongly associated with neurodegenerative disease (Postuma et al., 2015).

Parasomnia overlap disorder was first described in 1997 as a special form of parasomnia (Schenck et al., 1997) and is considered to be a variant of RBD (Dumitrascu et al., 2013). Patients with POD are predominantly male and younger than most idiopathic cases of RBD.

Clinically, we often encounter OSAHS accompanied by disturbed dreams or other parasomnias (Bugalho et al., 2017; Gabryelska et al., 2018; Huang et al., 2011), but OSAHS–comorbid POD is relatively rare. Most cases of OSAHS–comorbid POD are reported in individual cases (Soca et al., 2016). Here, we report a male patient who suffered from more than 6 years of disturbed dreams in which OSAHS and POD were diagnosed by PSG and in which his disturbed dreams almost disappeared following continuous positive airway pressure (CPAP) treatment. This report highlights and lists what is unique or novel about this case report.

## Case Description

A 46-year-old male patient (weight 71.5 kg; height 169 cm; body mass index 25.03 kg/m^2^; neck circumference 45 cm; modified Mallampati score 3) was referred to our sleep center with a 6-year history of vivid, disturbed dreams and 3-month history of repeated nocturnal episodes of violent and complex behaviors, clearly reflecting dream enactment with frequent dream recall. In these 3 months, due to the increased workload, he was under tremendous stress. During the episodes, the patient often screamed, fell from the bed, and injured himself. The nightmare episodes were reported to recur seven to eight times per month and the abnormal behavior during sleep to recur one or two times per month at the time of our evaluation.

The patient’s main complaint was disturbed dreams occurring almost every day, which would seriously affect his emotions. The dreams, which would involve losing his children, drowning, being chased, and fighting with people and other difficult scenarios, made him feel nervous and fearful. After getting up in the morning, he felt upset, drowsy, and dizzy. He could remember the contents of his dreams, but he could not remember the abnormal behaviors during sleep, such as sitting up suddenly and shouting. Brain magnetic resonance imaging (MRI) was unremarkable, and repeated 24 h electroencephalography (EEG) tests did not show any epileptic activity. He used to take clonazepam 2 mg at bedtime, but he felt worse. He denied taking any other medications.

In addition, the patient reported that he suffered from snoring during sleep every day for nearly 10 years, which often caused waking in the night, together with excessive daytime somnolence. More recently, the patient also reported low-energy levels and memory loss.

He denied smoking and did not present any relevant family history of seizures. His mother suffered from sleepwalking. There were no abnormalities found in his physical, laboratory, or neurological examinations. At neurologic examination, he was alert and oriented, with no speech impairment. His cranial nerves did not show any alterations. Results of motor and sensory examinations were normal in all four extremities. Coordination and gait were normal. The cranial computed tomography, MRI scans, and EEG were normal. Psychiatric examination showed a score of 9 on the Hamilton Anxiety Scale, 5 on the Hamilton Depression Scale, 13 on the Epworth sleepiness scale, and a Pittsburgh sleep quality index of 19.

**FIGURE 1:**
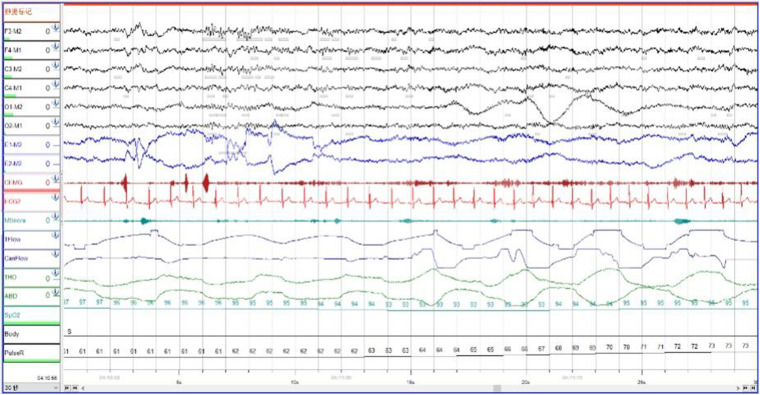
During Rem sleep, an excessive amount of tonic chin electromyogram activations was evident.

**FIGURE 2:**
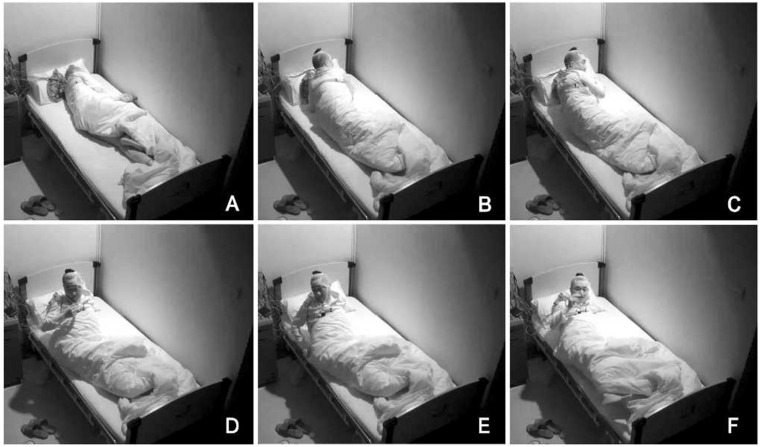
Complex behaviors were detected during N3 sleep through video. The specific movements are as follows: he suddenly sat up from N3 sleep stage with eyes opened widely, shouted, and aimlessly moved his limbs. **(A–F)** Indicates the order in which abnormal actions occur.

**FIGURE 3:**
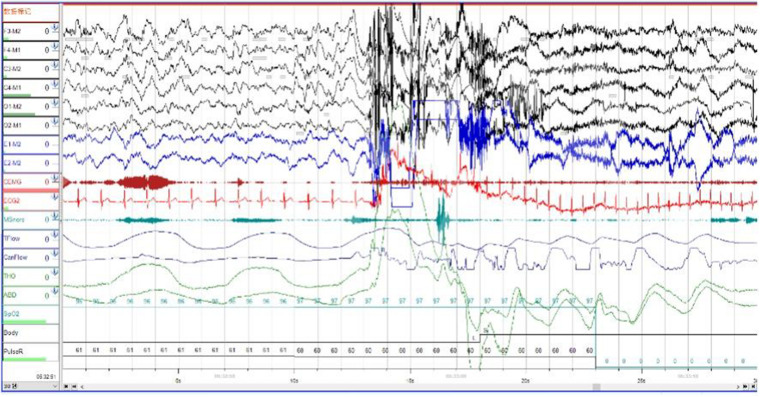
The patient’s heart rate rapidly increased from 60 to 105 beats/min with the increase in breathing rate. Brain wave changes from delta to alpha wave.

In order to exclude diseases such as seizures, we arranged PSG for patients. Nocturnal video PSG (v-PSG) revealed a sleep latency of 14 min, REM latency of 61 min, and sleep efficiency of 72.9%. Abnormal representation of the different sleep stages was also found, with increased N1% sleep period time (SPT) of 25.6%, decreased N2% SPT of 35.5%, N3% SPT of 18.2%, and REM% SPT of 20.8%. His sleep respiratory pattern was severely abnormal (apnea/hypopnea index of 40.1/h with average oxygen saturation of 95%) and showed a significant correlation with arousals. His leg movement during sleep (LM) index was 3.5/h. His arousal index was 20/h, and times of arousals were less in REM sleep (a total of eight times) than in NREM sleep (a total of 84 times). His respiratory-associated arousal index was 12.8/h, PLM-associated arousal index was 1.0, and spontaneous arousal index was 4.1/h (Table 1). Finally, an excessive amount of tonic chin electromyogram activations was evident during REM sleep (Figure 1), and complex behaviors were detected during N3 sleep through video. The specific movements are as follows: he suddenly sat up from N3 sleep stage with eyes opened widely and so on (Figure 2). Simultaneously, his heart rate rapidly increased from 60 to 105 beats/min with the increase in breathing rate. No EEG changes are seen during the event except for muscle artifact and movement artifact (Figure 3).

**TABLE 1:** Polysomnography report.

**Sleep summary**							
Lights out: 00:36:58				The latency to sleep onset: 14.0 min			
Lights on: 07:16:58				The latency to REM sleep onset: 61.0 min			
The total recording time: 400.0 min				Wake after sleep onset (WASO): 94.5 min			
The total sleep time (TST): 291.5 min							
**Sleep data**							
Stage	w		R	N1	N2		N3
Duration (min)	108.5		60.5	74.5	103.5		53.0
%Total sleep time			20.8	25.6	35.5		18.2
**Respiratory data**							
	OA		MA	CA	Apnea		Hypopnea
Number	56		0	16	72		123
Index (#/h TST)	11.5		0.0	3.3	14.8		25.3
Mean dur (s)	18.9		0.0	15.7	18.2		28.7
Max dur (s)					51.0		58.5
**Respiratory events**							
*REM/NREM*		REM		NREM		SLEEP	
*OA*		14		53		72	
Hypopnea		15		95		123	
A + H		29		148		195	
AHI (/h)		28.8		38.4		40.1	
RERAs		0		0		0	
RDI(/h)		28.76		38.4		40.1	

Combined with the clinical history and v-PSG findings that satisfied the International Classification of Sleep Disorders, third edition (ICSD-3) diagnostic criteria, we made a diagnosis of severe OSAHS and POD. After careful consideration of the therapeutic possibilities with the patient and his spouse, an agreement was made concerning therapy. Auto-set CPAP (APAP) was supplied for use at pressures ranging from 5 to 18 cm H_2_O to severe OSAHS, with an apnea/hypopnea index of 3.2/h after APAP treatment. When we followed up after 8 weeks of treatment, his abnormal behaviors, unpleasant dreams, snoring, and daytime hypersomnolence had been eliminated. Psychiatric examination showed a score of 5 on the Hamilton Anxiety Scale, 4 on the Hamilton Depression Scale, and 3 on the Epworth Sleepiness Scale and a Pittsburgh sleep quality index of 5.

Unfortunately, he refused to take PSG monitoring again during CPAP treatment. Nevertheless, his curative effect after treatment is amazing. The abnormal behavior and nightmares during his sleep almost disappeared. It can be considered that his symptoms of POD were symptomatic, not idiopathic. The complete eradication of POD symptoms following APAP treatment further confirmed our diagnoses of symptomatic POD, as had it been idiopathic POD, the treatment would have exacerbated or have not improved his symptoms of POD.

## Discussion

Obstructive sleep apnea hypopnea syndrome can trigger a variety of physical and psychological symptoms, including both parasomnias and seizures (Brunetti et al., 2017). It can also affect patients’ cognitive function (Gupta and Simpson, 2015; Kim et al., 2017; Krysta et al., 2017), work ability, and traffic safety (Hirsch Allen et al., 2015). The chronic intermittent airway collapse in patients with OSAHS presents sleep instability, including sleep fragmentation, CAP cycle increases (Halasz et al., 2004), and an increased homeostatic sleep drive (Howell, 2012; Brunetti et al., 2017), which can lead to an increased frequency to sleep parasomnias and seizures. For most, positive airway pressure treatment is the first-line treatment (Gottlieb and Punjabi, 2020), and inappropriate treatment may exacerbate OSAHS (Wang et al., 2018).

Here, we particularly emphasize the importance of distinguishing nocturnal seizures from parasomnias. Features supporting an epileptic etiology of paroxysmal events are (Salman et al., 2020) stereotyped nature of the spells; (Jehan et al., 2018) high frequency and tendency to cluster; and (Kapur et al., 2017) timing of the events (NREM parasomnias usually emerge from slow-wave sleep, which typically occurs within 2 h of sleep onset, whereas frontal lobe seizures may occur during any sleep stage but are common shortly after falling asleep). Nocturnal frontal lobe seizures can manifest as paroxysmal arousals (which consist of brief, sudden eye opening, head raising, or sitting up in bed; a frightened expression; and sometimes, vocalization) or nocturnal paroxysmal dystonia (which involves dystonic posturing and hypermotor phenomena and episodic nocturnal wanderings, which are longer in duration, 1–3 min, with associated stereotyped dystonic movements). Although it can be difficult to differentiate epileptic arousals from physiological arousals, sleep usually lightens after epileptic arousals, while people often return to the same sleep stage with brief physiological arousals/movements. Seizures cluster and occur throughout the night, often many times per night. Non-rapid eye movement parasomnias usually occur from deep sleep. Sleep terrors are usually distinguished by their strong associated autonomic features, including tachycardia and diaphoresis, and lack of recall the next day. Loud and prolonged screaming can occur, along with violent thrashing and bolting from bed in a terrified state. Certain people seem predisposed to having NREM parasomnia, and there is often a family history. Non-rapid eye movement parasomnia can be precipitated by sleep deprivation, stress, and other sleep disorders (e.g., sleep apnea). People are invariably confused during the event and are usually amnesic for the event (Boursoulian et al., 2012). No evidence of epilepsy was found in the patient in this article, and the various manifestations were supporting the diagnosis of parasomnias.

Parasomnias are dissociated sleep states which are partial arousals during transitions between wakefulness, NREM sleep, REM sleep, and their combinations (Howell, 2012). Multiple studies have shown that OSAHS is associated with parasomnias, including nightmares (BaHammam and Almeneessier, 2019), RBD, sleepwalking, and sleep terrors (Guilleminault et al., 2003; Haupt et al., 2013). A study of 84 children with recurrent or long-term sleepwalking showed that 58% had OSAHS. This shows that patients with OSAHS are more likely to suffer from parasomnias, and the symptoms of sleepwalking are significantly reduced after treatment of OSAHS (Guilleminault et al., 2003).

OSAHS provokes repeated cortical arousals. It also promotes sleep inertia leading to NREM parasomnias by impairing normal arousal mechanisms (Castelnovo et al., 2018). It has been ascertained that arousals are not isolated events but are basically endowed with a periodic nature expressed in NREM sleep by the cyclic alternating pattern (CAP). The sleep EEG CAP is an intrinsic oscillation, between periods of cortical arousal and quiescence throughout NREM sleep (Thomas, 2007). This oscillation provides the scaffolding for normal (e.g., delta bursts) and pathological (confusional arousal and SW events) NREM phenomena (Parrino et al., 2014). Functional significance of arousal in sleep, and particularly CAP, is to ensure the reversibility of sleep, without which it would be identical to coma. In this dynamic perspective, ongoing phasic events carry on the one hand arousal influences and on the other elements of information processing. The other function of arousals is tailoring the more or less stereotyped endogenously determined sleep process driven by chemical influences according to internal and external demands. In this perspective, arousals shape the individual course of night sleep as a variation of the sleep program (Halasz et al., 2004). Obstructive sleep apnea hypopnea syndrome patients have increased CAP measures (Korkmaz et al., 2018); therefore, they are more conditioned to suffer from nightmares and other parasomnias. Changes in the CAP, a biomarker of arousal instability in NREM sleep, are noted in NREM parasomnias (Parrino et al., 2012) and some neurodegenerative diseases (Melpignano et al., 2019).

Polysomnography in POD typically demonstrates NREM sleep instability in combination with a lack of REM sleep atonia (Schenck et al., 1997; Matos et al., 2016; Xu et al., 2019). Parasomnia overlap disorder linking RBD with disorders of arousal may serve to expand the concept of state-dependent motor dyscontrol (Schenck and Howell, 2019). If this motor dyscontrol occurs during NREM, the result is disorders of arousal; if it occurs during REM, the result is RBD (Schenck and Mahowald, 1996; Ferri et al., 2017; Hrozanova et al., 2019). The existence of a NREM–REM sleep disorder suggests that the basic abnormality of the motor parasomnias may consist of motor dyscontrol during sleep, with the affected sleep stage and the type of parasomnia being influenced by developmental, biological, and clinical factors (Hrozanova et al., 2019; Schenck and Howell, 2019).

Parasomnia overlap disorder represents an extreme breakdown of state boundaries in which there is a mixture, or rapid cycling, of NREM, REM, and wakefulness (Schenck and Mahowald, 2005; Castelnovo et al., 2018; Schenck and Howell, 2019). It is related to various nervous system diseases, mental diseases, and other sleep diseases, including narcolepsy, multiple sclerosis, Creutzfeldt–Jakob disease (Puligheddu et al., 2017), drug abuse, and sleep disordered breathing (Bugalho et al., 2017). In this case, POD symptoms were triggered by sleep disordered breathing. Studies have found that contrary to RBD, POD does not seem to be a risk factor for neurodegenerative diseases of α-synuclein (Dumitrascu et al., 2013; Schenck and Howell, 2013).

The pathogenesis of parasomnia remains unclear. It is presumed that damage to the awakening mechanism triggers the separation between the motor components of the awake state and the brain’s electrical activity during sleep (Kotagal, 2013). In NREM parasomnias, impaired arousal mechanisms and the persistence of sleep drive result in a failure of the brain to fully transition into wakefulness (Hrozanova et al., 2019). The transition between the three sleep states (awake, NREM sleep, and REM sleep) is not a quick and simple process; the release of various neurotransmitters must change before a specific state is clearly manifested (Howell, 2012). Recent research has shown that one or more combinations of these states may lead to unstable states, and awakening from these unstable states may lead to abnormal behavior, manifested as parasomnias (Castelnovo et al., 2018).

Patients with OSAHS often present at sleep centers because of nightmares (BaHammam and Almeneessier, 2019) and other parasomnias. Some symptoms of parasomnias are secondary to OSAHS (Goyal et al., 2018), and when OSAHS is effectively treated, the symptoms of parasomnia are also relieved (Carrasco et al., 2006). Symptomatic parasomnia is different from idiopathic parasomnia in pathophysiology, complications, and treatment. For example, idiopathic RBD is generally treated with clonazepam (Howell, 2012), while the use of clonazepam in patients with symptomatic RBD (related to OSAHS) is likely to aggravate the symptoms of OSAHS (Wang et al., 2018). Therefore, PSG should be performed for patients with suspected parasomnia to distinguish idiopathic parasomnia from symptomatic (related to OSAHS), which is more conducive to disease treatment.

## Conclusion

Abnormal behaviors during sleep exhibit a myriad of symptoms. Their underlying diseases are also diverse, which include NREM-/REM-related parasomnias, epilepsy, and mental disorders. Non-rapid eye movement parasomnias and epilepsy can sometimes be difficult to distinguish, especially on history alone. The patient we reported in this article is an OSAHS patient with nightmares as the main complaint accompanied by POD symptoms (RBD and night terror). The symptoms of POD almost completely disappeared after CPAP treatment. Studies have found that in a large proportion of adults (Bjorvatn et al., 2010; Hrozanova et al., 2019) and children (Krysta et al., 2017) who meet the diagnostic criteria for chronic NREM parasomnia, it is induced by OSAHS, especially when the characteristics of parasomnia are unusual. Physicians should determine whether it is induced by OSAHS as this will affect subsequent treatment options. When they coexist, successful treatment of OSAHS can usually resolve parasomnia symptoms (Schenck and Mahowald, 2008). Since abnormal behaviors during sleep may severely affect a patient’s quality of life, giving an early and accurate diagnosis of the underlying disease (by analyzing v-PSG data during the manifestation of abnormal behaviors during sleep) is of great importance. Therefore, v-PSG should be routinely performed for patients with parasomnias and other abnormal sleep behavior suspected of OSAHS. When encountering abnormal sleep behavior patients with comorbid OSAHS clinically, if the abnormal sleep behavior is first treated, OSAHS will often be aggravated. Therefore, the primary goal of treatment for these patients is to solve OSAHS first. In the future, we can pay attention to the collection of similar cases in clinical work, so as to provide better medical services for these patients.

## Data Availability Statement

The original contributions presented in the study are included in the article/Supplementary Material, further inquiries can be directed to the corresponding author.

## Ethics Statement

The studies involving human participants were reviewed and approved by Ethics Committee of Tianjin Anding Hospital. The patients/participants provided their written informed consent to participate in this study. Written informed consent was obtained from the individual(s) for the publication of any potentially identifiable images or data included in this manuscript.

## Author Contributions

JL contributed to the conception and design of the manuscript. YS wrote the first draft of the manuscript. YS and XZ wrote the sections of the manuscript. All authors contributed to manuscript revision and read and approved the submitted version.

## Conflict of Interest

The authors declare that the research was conducted in the absence of any commercial or financial relationships that could be construed as a potential conflict of interest.

## Abbreviations

OSAHS: obstructive sleep apnea hypopnea syndrome
POD: parasomnia overlap disorder
RBD: REM sleep behavior disorder
v-PSG: video polysomnography
CAP: cyclic alternating pattern
NREM: non-rapid eye movement sleep
REM: rapid eye movement sleep
APAP: auto-set continuous positive airway pressure.
